# NDH expression marks major transitions in plant evolution and reveals coordinate intracellular gene loss

**DOI:** 10.1186/s12870-015-0484-7

**Published:** 2015-04-11

**Authors:** Tracey A Ruhlman, Wan-Jung Chang, Jeremy JW Chen, Yao-Ting Huang, Ming-Tsair Chan, Jin Zhang, De-Chih Liao, John C Blazier, Xiaohua Jin, Ming-Che Shih, Robert K Jansen, Choun-Sea Lin

**Affiliations:** Department of Integrative Biology, University of Texas at Austin, Austin, TX USA; Agricultural Biotechnology Research Center of Academia Sinica, Agricultural Technology Building, No. 128, Sec. 2, Academia Road, Nankang, Taipei, 115 Taiwan; Institute of Biomedical Sciences, National Chung Hsing University, Taichung, Taiwan; Department of Computer Science and Information Engineering, National Chung Cheng University, Chia-Yi, Taiwan; Institute of Botany, Chinese Academy of Sciences, Beijing, 100093 China; Department of Biological Science, Biotechnology Research Group, King Abdulaziz University, Jeddah, 21589 Saudi Arabia

**Keywords:** Streptophyta, NAD(P)H dehydrogenase, Transcriptomics, Cyclic electron flow, Sig4

## Abstract

**Background:**

Key innovations have facilitated novel niche utilization, such as the movement of the algal predecessors of land plants into terrestrial habitats where drastic fluctuations in light intensity, ultraviolet radiation and water limitation required a number of adaptations. The NDH (NADH dehydrogenase-like) complex of *Viridiplantae* plastids participates in adapting the photosynthetic response to environmental stress, suggesting its involvement in the transition to terrestrial habitats. Although relatively rare, the loss or pseudogenization of plastid NDH genes is widely distributed across diverse lineages of photoautotrophic seed plants and mutants/transgenics lacking NDH function demonstrate little difference from wild type under non-stressed conditions. This study analyzes large transcriptomic and genomic datasets to evaluate the persistence and loss of NDH expression across plants.

**Results:**

Nuclear expression profiles showed accretion of the NDH gene complement at key transitions in land plant evolution, such as the transition to land and at the base of the angiosperm lineage. While detection of transcripts for a selection of non-NDH, photosynthesis related proteins was independent of the state of NDH, coordinate, lineage-specific loss of plastid NDH genes and expression of nuclear-encoded NDH subunits was documented in Pinaceae, gnetophytes, Orchidaceae and Geraniales confirming the independent and complete loss of NDH in these diverse seed plant taxa.

**Conclusion:**

The broad phylogenetic distribution of NDH loss and the subtle phenotypes of mutants suggest that the NDH complex is of limited biological significance in contemporary plants. While NDH activity appears dispensable under favorable conditions, there were likely sufficiently frequent episodes of abiotic stress affecting terrestrial habitats to allow the retention of NDH activity. These findings reveal genetic factors influencing plant/environment interactions in a changing climate through 450 million years of land plant evolution.

**Electronic supplementary material:**

The online version of this article (doi:10.1186/s12870-015-0484-7) contains supplementary material, which is available to authorized users.

## Background

Key innovations have facilitated novel niche utilization, such as the movement of the algal predecessors of land plants into terrestrial habitats [1] where fluctuations in light intensity, ultraviolet radiation and water limitation required a number of adaptations [1,2]. Among them, mechanisms that permitted more refined control of the light reactions of photosynthesis may have evolved. The NDH (NADH dehydrogenase-like) complex of plant and algal plastids, which participates in cyclic electron flow (CEF), was initially identified through its homology to the mitochondrial respiratory complex I [3], then shown to be more similar to the NDH-1 pathway of extant cyanobacteria [4,5].

During the light reactions of photosynthesis in cyanobacteria, algae and plants, photons excite pigment/chlorophyll molecules in photosystem II (PSII) at the stromal face of the chloroplast thylakoid membrane. This light energy, in the form of water derived electrons, is ultimately relayed to photosystem I (PSI) via the intermediate carriers of the electron transport chain to drive proton translocation across the membrane and into the thylakoid lumen. The resulting proton gradient is coupled to ATP synthesis via ATP Synthase with the concomitant reduction of NADP to yield NADPH by ferredoxin-NADP reductase. This process is referred to as linear electron flow and/or transport (LEF). While LEF is the primary pathway for conversion of photons into storable energy, CEF occurs exclusively around photosystem I and contributes to generation of the proton gradient. However the electrons that reduce the plastoquinone (PQ) in CEF are recycled directly from ferredoxin (Fd) allowing for the generation of ATP without the production of NADPH. The resulting high pH differential across the thylakoid membrane induces non-photochemical quenching (NPQ) allowing the dissipation of excess electrons under potentially unfavorable growth conditions including fluctuating, high intensity light, low CO_2_ concentration or drought stress [6,7].

Two independent pathways of CEF have been characterized across Streptophyta, the lineage that includes charophyte algae and land plants [6,8]. The genomes of extant cyanobacteria encode the core components of the NDH complex and a pathway for CEF that is sensitive to antimycin A (AA) [9] as has been elucidated in land plants [6]. Sequencing of the *Klebsormidium flaccidum* genome, a streptophyte alga, revealed genes encoding both a functional NDH, with subunit genes in the plastid and nuclear genomes, and components of the nuclear encoded PGR5-dependent (Proton Gradient Regulation5; AA sensitive) pathway of CEF [1].

While both pathways clearly originated in the ancestor of plastids, and both rely on Fd-dependent reduction of PQ, the PGR5-dependent pathway is the main contributor to the pH differential and ultimately ATP generation in CEF [6]. This may be the reason for its ubiquity among the photosynthetic organisms studied to date, whereas NDH CEF has been lost in all photosynthetic lineages examined other than Streptophyta [1,8]. The two pathways are encoded by non-overlapping gene sets and are therefore predictably mechanistically distinct [10].

Cyclic electron flow is essential to efficient photosynthesis and complete inhibition of CEF severely affects LEF *in vivo* [10]; however, the specific role of NDH in CEF remains obscure. Studies support NDH involvement in redox balancing under abiotic stress as it appears to mediate electron flow from stromal reductants to PQ [11-13]. Phenotypes, such as photoinhibition, reduced growth rate and the loss of NPQ induction, are severe where both CEF pathways are restricted [10-14], pronounced when the PGR5-dependent pathway is impaired and subtle when only NDH expression is ablated [5,10,15].

Under favorable growth conditions disruption of NDH has little effect. However CO_2_ limitation, extended exposure to low temperatures and high or low light intensities revealed mildly deleterious phenotypes. *Oryza sativa crr6* (required for NDH complex biogenesis) mutants lacked the post illumination increase in chlorophyll fluorescence that is a hallmark of Fd-dependent PQ reduction, and exhibited mild inhibition of all photosynthetic parameters measured and a diminution in plant biomass when grown at 20°C [16]. The disruption of plastid *ndhB* in *Marchantia polymorpha* resulted in a PQ pool that was significantly more reduced at low light intensities relative to the wild type [12]. *Nicotiana tabacum* inactivated for plastid encoded NDH-B showed a reduction in transient chlorophyll fluorescence following actinic illumination, but otherwise performed normally [5]. Repeated, brief exposure to strong light, however, resulted in photoinhibition of PSII and irreversible chlorosis in the same transformed line whereas wild type leaves exposed to the same treatments recovered [13].

Like many plastid-localized, multi-subunit complexes, both plastid- and nuclear-encoded proteins assemble to form the NDH complex [17]. Although relatively rare, the loss or pseudogenization of plastid NDH genes is nonetheless noted across diverse lineages of photoautotrophic seed plants with examples found among gymnosperms [18,19], and both monocot [20-24] and eudicot lineages of angiosperms [25]. As in the preceding experimental examples, these species appear unaffected by the lack of plastid NDH gene expression and several authors have suggested that the missing NDH constituents may have been functionally transferred to the nucleus [18-22,25,26].

Based on phenotypes of mutants/transgenics, NDH function appears to be dispensable under favorable growth conditions; however, the plastid-encoded genes for NDH subunits are conserved across the phylogeny of Streptophyta suggesting a strong selective advantage in retention of NDH function [1]. This study uses large-scale transcriptomic and genomic analyses to evaluate the persistence and loss of NDH expression. Nuclear expression profiles showed that there have been acquisitions of novel NDH genes at key transitions throughout 450 million years of land plant evolution. In the clades where the NDH genes are missing from the plastid genome, no evidence of intracellular horizontal gene transfer of NDH genes was detected. Rather coordinate, lineage-specific loss of expression of nuclear-encoded NDH subunits confirmed the independent loss of NDH in select taxa across the seed plant phylogeny.

## Results and discussion

**Figure 1:**
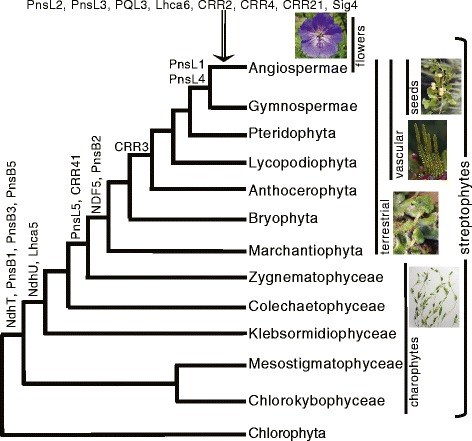
Accretion of NDH complexity through streptophyte evolution. Plotted at the nodes are novel genes that have no known homologue in extant cyanobacteria. Gene acquisitions were placed at the node to indicate first appearance regardless of putative, subsequent losses. Note that the PnsB3 transcript was detected in one species among the three Chlorophyta included (Additional file 1).

To evaluate the distribution and timing of changes in the NDH gene complement across land plants a subject database comprising nuclear transcriptomes of photoautotrophic Streptophyta species (listed in Additional file 1) was queried with *Arabidopsis* NDH-related coding sequences and the results of the survey mapped in a phylogenetic context. Queries included constituents of each of the four major subunits containing nuclear proteins, the Lhca (light harvesting complex associated) proteins involved in tethering NDH to PSI during supercomplex formation, assembly and accessory proteins, *ndhF* transcription factor Sig4 and proteins involved in maturation and editing of NDH transcripts in plastids (see Additional file 1 for a complete list). The core constituents of NDH are encoded by genes derived from the common cyanobacterial ancestor of all photosynthetic eukaryotes, 11 genes encoded in the plastid (*ndhA-K*) and five in the nucleus (*ndhL-S*) [17]. Additional related sequences (subunits and auxiliary proteins) were acquired through time and demonstrate an accretion in complexity within NDH, notably at the split between the chlorophyte algae and streptophytes, between charophytes and terrestrial plants, at the origin of seed plants and the base of angiosperms (Figure 1). The more recent gene acquisitions portray an increasing requirement for coordinated control of plastid-encoded gene expression. The majority of gains occurred prior to the diversification of angiosperms and include *ndhF* transcription factor Sig4, and PPR proteins involved in processing of plastid NDH polycistrons and RNA editing (Figure 1; Additional file 1). The co-emergence of Lhca6 at the base of angiosperms could be related to the apparent requirement for tightly controlled expression of subunits. Supercomplex formation with PSI is thought to positively influence stability of NDH in flowering plants, although a role in NDH assembly has not been discounted [27]. Nuclear control of plastid gene expression may be a means to regulate the stoichiometry of PSI:NDH subunits for optimal efficiency. Whole genome duplication, which occurred in virtually all angiosperms [28], may have been important in generating nuclear substrate sequences for sub- or neo-functionalization yielding the more complex NDH system. Greater control and efficiency of the CEF system, among many other innovations (i.e. [29-31]), may have played a role in the eventual angiosperm radiation into virtually every terrestrial ecosystem.

**Figure 2:**
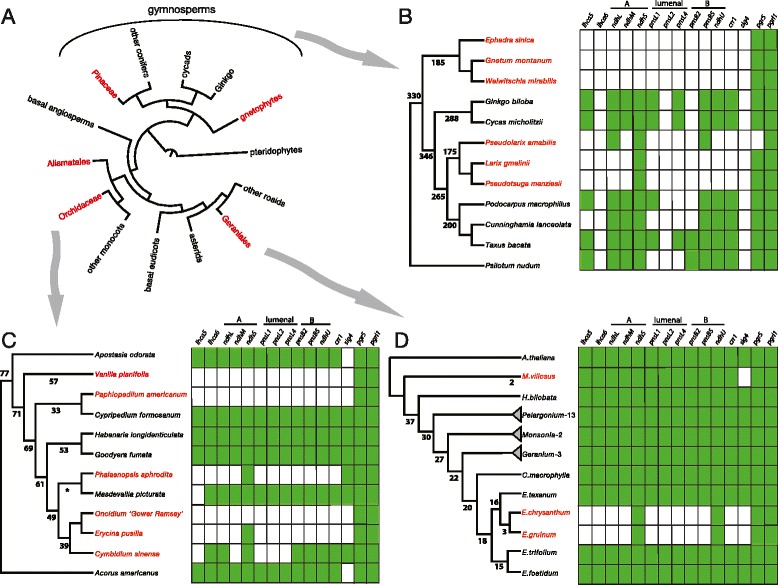
Distribution and timing of plastid and nuclear NDH gene loss across seed plants. Representation of land plant relationships **(A)**. Groups where photosynthetic taxa have lost/pseudogenized plastome NDH genes are indicated with red font; arrows indicate the relevant cladogram for each subgroup. **(B-D)** Cladograms showing divergence times and lineage specific loss of nuclear transcripts encoding NDH proteins. ‘A, lumenal and B’ refer to subunits in the NDH complex. Green shading indicates detection of the nuclear transcript for each gene. Numeric values indicate the divergence time (MYA: million year ago) for each lineage. In *C*, the asterisk indicates a questionable branch discussed in the text. In *D*, the number after the generic name indicates the number of species included in the analysis with identical patterns with regard to NDH expression. Orchid and Geraniales relationships are represented by plastid 12-gene maximum likelihood trees (see Methods); gymnosperm phylogeny is based on two plastid genes, *rbcL* and *matK*.

Despite near ubiquity, a number of exceptional cases exist among photosynthetic species where the NDH genes have been pseudogenized or are entirely missing from plastid genomes (Figure 2A). Among plastomes of Viridiplantae, the loss of NDH genes has been reported for all species of Gnetophyta and Pinaceae, several species in the Orchidaceae and in the long-branch clade within the genus *Erodium* (Geraniaceae). It appears that another species in the Geraniales, *Melianthus villosus*, is undergoing NDH loss as four of its plastome-encoded NDH genes are pseudogenes [32]. Plastid NDH gene loss was recently revealed in the monocot family Hydrocharitaceae, which harbors at least three species of aquatic angiosperms that have undergone NDH disruption (*Najas Vallisneria*, *Thalassia*) with two further losses likely among the marine ‘seagrasses’ in the order Alismatales (*Posidonia*, *Amphibolis*) [23,24].

Of the non-NDH plastome genes that have been lost or pseudogenized, several have been detected in the nuclear genome where their expression is regulated by nuclear factors and subsequent products targeted back to plastids. Although several groups have suggested that plastid NDH pseudogenes may be functionally represented by nuclear sequences [18-22,25], no reports describe a functionally transferred, nuclear NDH gene of recent plastid origin. A number of nuclear-encoded NDH subunits and accessory proteins have been elucidated [6,8], which are targeted to the plastid where they assemble with plastome-encoded subunits at the thylakoid membrane. Figures 2B-D present an abbreviated graphical overview of the pattern of NDH nuclear gene loss. In all investigated lineages where the plastid sequences have been lost, no transcripts were detected that could represent recently transferred plastid NDH genes. Furthermore, transcripts reporting expression of nuclear-encoded NDH subunits are, to varying degrees, absent (Figure 2B-D, Additional file 1). To confirm that the lack of nuclear NDH transcripts is exclusive to NDH genes and not a more general phenomenon and to demonstrate that there were sufficient data to detect such transcripts were they present, transcriptomes of plastid NDH-deleted species and those that retain NDH in the plastome were queried for the presence of transcripts encoding members of gene families to which NDH subunits belong. Regardless of the state of the NDH system nearly all searched non-NDH transcripts inherent to photosynthesis and plastid respiration were detected (Additional file 2). In a very few cases where data were mined from public databases, expected transcripts (i.e. PGR5) were not detected. Overall the mined data yielded findings highly similar to those found using the data produced for this study, the collection of which was standardized according to Zhang et al. [33]. The downloaded transcriptomes varied broadly in terms of tissue type, library construction, sequence length and read depth, however they were of sufficiently high quality to detect most if not all genes searched for this study. The use of *Arabidopsis* reference sequences may have also allowed some transcripts to go undetected due to high levels of divergence between the reference and the taxa examined.

Indeed, the ‘patchy’ signal detected for *lhca5* (Additional file 1) prior to its consistent presence in angiosperms could be a result of the caveats described above. For this study the parameters used to define a transcript as present or absent, as described in the methods, were set *a priori*, as was the decision to report the first incidence of detection regardless of subsequent lack of detection in more recently diverged lineages. The chlorophyte algae *Clamydomonas reinhardii* and *Ostreococcus tauri*, among others, are thought to contain a gene encoding Lhca5 (GenBank, ABD37907 and AAY27547.1), however the transcript was not detected using the present data mining strategy. Of course these species are known to lack NDH and likely their *lhca5* sequences are not similar enough to be detected using *Arabidopsis* queries. Even in more closely related groups, i.e. gymnosperms and angiosperms, there is a margin for error although calls of presence or absence are expected to be more reliable between more recently diverged groups. The *lhca5* transcript was detected in seven of 11 gymnosperm species that are competent for NDH CEF (Additional file 1).

The presence of the AA-sensitive pathway described by Arnon et al. [34], the predominant pathway for CEF in plants, was inferred by the identification of nuclear transcripts encoding PGR5 and PGRL1 (A/B) in nearly all species surveyed (Figure 2B-D; Additional file 1). Although wholesale loss of nuclear-encoded NDH gene expression is typical of the taxa in which plastome NDH genes are absent, there is some variation across this select group. One ancestral NDH subcomplex A gene that is retained across the majority of taxa except gnetophytes is the nuclear gene encoding NdhS (Crr31), responsible for high affinity binding of ferredoxin [11] (Figure 2; Additional file 1). In experiments where this function is ablated, NDH complex formation is concomitantly lost. However NdhS accumulated in the thylakoid membranes of mutants that do not express NDH suggesting this protein may have an additional function in plastids [11].

The gene encoding the thylakoid immunophilin PnsL5 (CYP20-2) lacks a cyanobacterial homolog and is nearly ubiquitous across the species surveyed, apparently arising at the node leading to Zygnematophycaeae, the proposed sister lineage to land plants (Figure 1, Additional file 1). Although PnsL5 has been identified in NDH and NDH-PSI [35] preparations as a lumenal subcomplex protein and its expression was strongly reduced in NDH mutants with defects in the hydrophobic domains, mutants lacking PnsL5 expression were not affected in complex stability or NDH activity [36]. There remains some question regarding its status as a bona fide subunit of NDH; while PnsL5/CYP20-2 may indeed be an NDH-associated protein this isomerase has demonstrated roles in gibberillic acid and brassinosteroid signaling in *Triticum* and *Arabidopsis*, respectively [37,38]. Given that phytohormone signaling has been proposed as an adaptation facilitating colonization of land by plants [30,39], the emergence of PnsL5/CYP20-2 in the sister lineage to land plants, tempts speculation of a dual role for this protein in the early adaptation to land.

A phylogenetic context permits evaluation of the distribution of retention/loss as a function of evolutionary time and thus the estimation of the number of independent losses that have occurred. For example within gymnosperms there are two distinct groups lacking plastid NDH genes; all sampled gnetophytes and Pinaceae. There has been considerable controversy regarding the phylogenetic position of gnetophytes among gymnosperms with several different placements supported by different data sets (i.e. gnepine, gnetifer, anthopyte, gnetophytes sister to all other gymnosperms) [40-45]. Accordingly, interpretation of the pattern of NDH gene loss will be affected by which of these hypotheses is correct. The tree shown in Figure 2B is based on limited taxon sampling and two plastid genes, *rbcL* and *matK*, and reflects the relationships identified in Lee et al. [46], placing gnetophytes as sister to all other gymnosperms. The complete excision of all plastid NDH genes in *Gnetum* and *Ephedra* and a single detectable pseudogene in *Welswitchia* [47,48] suggest that the loss in Pinaceae may be more recent as these plastomes retain many more NDH pseudogenes [18]. If indeed the relationships inferred by Lee et al. are correct, then two independent losses would be appropriately assigned. Alternatively, if gnetophytes are sister to Pinaceae, then the most parsimonious interpretation is of a single loss. Nonetheless, these events are relatively ancient (Figure 2B) and both groups have similarly lost function of all plastid genes for NDH along with nuclear NDH gene expression with the exception of *PnsL5* and, at least for the Pinaceae species, *ndhS* (Additional file 1). As seen in Orchidaceae, however, events interpreted as synapomorphic may in fact represent independent events whose signals have been obscured through evolutionary time. This phenomenon is well illustrated by the repeated, independent loss of the plastome *infA* and *accD* genes among others [49].

Among the Orchidaceae four independent losses of NDH are indicated (Figure 2C). However the 12-gene phylogeny presented here is limited to the 11 species for which both plastome and transcriptome data are available and differs somewhat in topology from trees incorporating hundreds of species. Expanded phylogenies have placed *Masdevallia* outside the clade of higher epidendroids that it groups with here [50]. This alternative placement predicts three independent NDH losses among orchids. Contrary to the suggestion that Sig4 evolved after the diversification of eudicots [51], *sig4* transcripts were detected among the orchid species that retain NDH function as well as in *Amborella* (Additional file 1).

Recent loss of plastid NDH genes is observed in the long- branch clade (LBC) of *Erodium*. Losses have been confirmed in three among 13 species through complete plastome sequencing; losses in all remaining species were inferred through PCR survey for the *ndhD* gene [25] suggesting that the loss occurred prior to the diversification of the clade (3 MYA). Within the Geraniales there appears to be an even more recent loss in the genus *Melianthus* where four NDH pseudogenes have been identified in the plastome [32]. This clade is believed to have diversified approximately 2 MYA and retains some apparently translatable plastome NDH sequences. Probing the *M. villosus* transcriptome revealed that while most NDH related transcripts were detected, the *ndhF* transcription factor Sig4 is transcribed as a pseudogene (Figure 2D).

## Conclusions

These data allow insight into the distribution and timing of NDH gene loss across seed plants and suggest at least limited dispensability of NDH function. In no case were functional nuclear copies of plastome NDH sequences identified but rather the degradation and loss of nuclear-encoded interacting proteins was revealed. The broad phylogenetic distribution of NDH loss and the subtle phenotypes of mutants suggest a high propensity for gene loss and may be indicative of its limited biological significance in contemporary plants [52]. On an evolutionary time scale, however, climate fluctuates in virtually all habitats such that NDH function may be intermittently significant. While NDH activity appears dispensable under favorable conditions there may be sufficiently frequent episodes of abiotic stress affecting terrestrial habitats to allow the retention of NDH activity throughout land plant evolution. In fact, the accumulation of RNA editing sites in plastid NDH genes has been interpreted as arising from such recurrent “dispensability and rescue” of NDH activity [8].

The timing of changes in the NDH gene complement supports the hypothesis that the NDH system, among other adaptations, has been involved in the transition to terrestrial habitats and possibly other key innovations throughout 450 million years of land plant evolution. Given the present outlook on climate change a deeper understanding of the genetic factors influencing the adaptation of land plants to novel conditions will inform management programs.

## Methods

### Plant materials

Tissue samples for species of Geraniales were harvested from the living collection at University of Texas at Austin. All specimens have been vouchered and deposited in the UT Plant Resources Center (TEX-LL). Voucher numbers are listed in Additional file 3.

*Apostasia odorata* was collected from Yunnan, China. *Cypripedium formosanum* was obtained from highland experimental farm, National Taiwan University in Mei-feng, Taiwan. Three commercial Orchidaceae species were purchased from a local grower in Taiwan, two Epidendroideae (*Masdevallia picturata* and *Erycina pusilla*) and one Orchidoideae (*Goodyera fumata*). These three species were grown in the greenhouses at Academia Sinica, Taipei, and National Chung Hsing University, Taichung, Taiwan.

### RNA isolation, transcriptome sequencing and assembly

Total RNA from newly emerged leaves of 26 species in Geraniales and four tissues (emergent and expanded leaves, roots and flowers) of *Pelargonium* x *hortorum* was isolated and cDNA libraries constructed following the protocols described in Zhang et al. [33]. Transcriptome sequencing was performed on the Illumina HiSeq™ 2000 platform (Illumina, San Diego, CA). For Orchidaceae, total RNA was extracted from young leaves by TRIzol® reagent (Life-Technologies, Taipei City, Taiwan). Agilent 2100 Bioanalyzer (Agilent Technologies, Taipei City, Taiwan) was used to confirm total RNA quality. Six paired-end RNA-Seq libraries (*A. odorata, C. formosanum, M. picturata, G. fumata, E. pusilla* and *H. longidenticulata*) were constructed using the Illumina TruSeq™ Stranded mRNA HT, insert size was 300 bp. The libraries were sequenced on Illumina NextSeq™ 500 paired-end system using a NextSeq™ 500 Mid output kit (300 cycles; Illumina). Sequence data of Geraniales and the five species of orchid were preprocessed and assembled as described in Zhang et al. [33]. Transcriptome data for the remaining species examined were downloaded from the NCBI Sequence Read Archive (SRA, Additional file 1). Reads were assembled with Trinity release 2013/11/10 (http://sourceforge.net/projects/trinityrnaseq/) using the parameters “--JM 100G --full_cleanup --min_contig_length 200 --CPU 24” on a 24-core 3.33 GHz linux work station with 1 TB memory at the Texas Advanced Computing Center (TACC, http://www.tacc.utexas.edu/).

### Identification of nuclear-encoded *ndh* genes

The protein sequences of reference genes of *Arabidopsis* were downloaded from TAIR [53]; accession numbers are given in Additional files 1 and 2. Reference gene sequences were aligned to the transcriptome assemblies using TBLASTN with e-value 1e-5 to extract the nuclear encoded candidate genes of the NDH complex (See Additional file 4). The candidate genes were then aligned to the *Arabidopsis* transcriptome using BLASTX with default settings, and the gene was considered present if the top hit was the corresponding reference gene. If the top hit was not the corresponding gene, manual inspection of these candidate genes was performed to resolve potential chimeric assembly problems and the candidate genes were confirmed again with BLASTX. All newly generated sequences have been submitted to NCBI GenBank, accession numbers are in Additional file 4.

### Phylogeny construction

The phylogenies of 11 species of orchids plus *Acorus* and 26 species of Geraniales plus *Arabidopsis* were generated by RAxML with parameters “raxmlHPC-PTHREADS-SSE3 -f a -x 12345 -p 12345 -T 12 -m GTRGAMMAI -N 100” using 12 plastid genes (*atpA*, *atpB*, *atpI*, *ccsA*, *cemA*, *matK*, *petA*, *rbcL*, *rpoB*, *rpoC1*, *rpoC2*, *rps2*), and bootstrap values were generated using RAxML with 100 replicates and the above settings. The gymnosperm phylogeny of 11 species plus *Psilotum* was generated by the same parameters except that two sequences, *matK* and *rbcL* were employed. For Figure 1 and 2A, trees were drawn manually based on relationships depicted in [54] and [45,46].

### Divergence time estimation

Divergence time estimates were derived from previous studies of gymnosperms [55,56], Orchidaceae [50] and Geraniales [57,58].

## Availability of supporting data

Phylogenetic datasets are available in Dryad Digital Repository (http://dx.doi.org/10.5061/dryad.h9m07). Sequence data are available in the Short Read Archive at GenBank, http://www.ncbi.nlm.nih.gov/sra; accession numbers are listed in Additional files 1, 2, 4.

## Additional files

Additional file 1:
**Expanded survey of nuclear encoded NDH transcripts.** This table includes data for 106 species, both generated in this study and downloaded from a number of sources. Additional file 2:
**Detection** ***psbP, psbQ, lhca*** **, FKBP and PGR gene family transcripts in gymnosperms, Orchidaceae and Geraniales.** This table includes data for 50 species as indicated in the title and reports the expression of nuclear genes unrelated to NDH. Additional file 3:
**Voucher information for 26 species of Geraniales.** University of Texas Plant Resources Center voucher numbers for Geraniales specimens used in this study. Additional file 4:
**Accession numbers for all specific transcript sequences generated in this study.** Collection of GenBank accession numbers for all new plastid genome and individual nuclear transcript sequences reported in this study.

## Abbreviations

LEF: Linear electron flow
CEF: Cyclic electron flow
PSI: Photosystem I
PSII: Photosystem II
AA: Antimycin A
NDH: Plastid NAD(P)H dehydrogenase-like complex
MYA: Million years ago
Lhca: Light harvesting complex genes/proteins
LBC: Long branch clade (as in *Erodium*)
