# Supplementary material for: NDH expression marks major transitions in plant evolution and reveals coordinate intracellular gene loss
Source: BMC Plant Biol. 2015 Apr 11;15:100. doi: 10.1186/s12870-015-0484-7 (PMC4404220; doi:10.1186/s12870-015-0484-7)
Supplement: Additional file 3: — Voucher information for 26 species of Geraniales. University of Texas Plant Resources Center voucher numbers for Geraniales specimens used in this study. [file 12870_2015_484_MOESM3_ESM.pdf]

Additional file 3: University of Texas at Austin Plant Resources Center (TEX-LL) voucher information for 26 species of Geraniales.

Species	TEX-LL Voucher #
California macrophylla	CB G1030
Erodium chrysanthum	CB G1039
Erodium cheilanthifolium	Weng G1032
Erodium gruinum	CB G1042
Erodium texanum	CB G1018
Erodium trifolium	Weng G1042
Geranium incanum	JZ G1001
Geranium maderense	Weng G1046
Geranium phaeum	Weng G1036
Hypseocharis bilobata	Weng G1041
Melianthus villosus	CB G1032
Monsonia emarginata	Weng G1033
Monsonia marlothii	Weng G1075
Pelargonium australe	Weng G1002
Pelargonium citronellum	Weng G1006
Pelargonium cotyledonis	Weng G1004
Pelargonium dichondrifolium	Weng G1010

Species	UTPRC Voucher #
Pelargonium echinatum	Weng G1018
Pelargonium fulgidum	Weng G1026
Pelargonium incrassatum	Weng G1009
Pelargonium myrrhifolium	CB G1006
Pelargonium nanum	CB G1015
Pelargonium tetragonum	Weng G1007
Pelargonium transvalenese	CB G1005
Pelargonium x *hortorum*	Weng G1003
Pelargonium exstipulatum	Weng G1020
